# Biogeography of the cosmopolitan terrestrial diatom *Hantzschia amphioxys *sensu lato based on molecular and morphological data

**DOI:** 10.1038/s41598-021-82092-9

**Published:** 2021-02-19

**Authors:** Yevhen Maltsev, Svetlana Maltseva, John Patrick Kociolek, Regine Jahn, Maxim Kulikovskiy

**Affiliations:** 1 К.A. Timiryazev Institute of Plant Physiology RAS, IPP RAS, Moscow, Russia 127276; 2 Museum of Natural History and Department of Ecology and Evolutionary Biology, University of Colorado, Boulder, CO 80309 USA; 3 Botanischer Garten und Botanisches Museum Berlin, Freie Universität Berlin, Königin-Luise-Str. 6-8, 14195 Berlin, Germany

**Keywords:** Phylogeny, Biogeography

## Abstract

Until now, the reported diversity of representatives from the genus *Hantzschia* inhabiting soils from different parts of Eurasia was limited to the few species *H. amphioxys*, *H. elongata* and *H. vivax* and some of their infraspecific taxa. We have studied the morphology, ultrastructure and phylogeny of 25 soil diatom strains, which according to published description would be assigned to “*H. amphioxys* sensu lato” using 18S rDNA, 28S rDNA and *rbc*L. We show that strains are made up of seven different species of *Hantzschia*, including five new for science. Five strains were identified as *H. abundans*. This species has a slight curvature of the raphe near its external proximal ends. Four of the examined strains were represented by different populations of *H. amphioxys* and their morphological characteristics fully correspond with its isolectotype and epitype. The main specific features of this species include 21–25 striae in 10 μm, 6–11 fibulae in 10 μm, 40–50 areolae in 10 μm and internal proximal raphe endings bent in opposite directions. *H*. *attractiva* sp. nov., *H*. *belgica* sp. nov., *H*. *parva* sp. nov., *H*. *pseudomongolica* sp. nov. and *H*. *stepposa* sp. nov. were described based on differences in the shape of the valves, significant differences in dimensions, a lower number of striae and areolae in 10 μm and the degree and direction of deflection of the internal central raphe endings. Based on the study of the morphological variability and phylogeny of soil *Hantzschia*-species from different geographical locations we conclude that while some species such as *H. amphioxys* are truly cosmopolitan in their distributions, some sympatric populations of pseudocryptic taxa exist in the Holarctic.

## Introduction

Diatoms are one of the most widespread group of unicellular algae, being reported from all types of water bodies and from all continents^1^. Currently, two hypotheses about freshwater diatom distribution are being discussed; one suggesting that all or most diatom species have a cosmopolitan distribution^2,3^ and the second that restricted geographic distributions and even endemicity are more common^4,5^. On the one hand, capacity for dispersal, wide environmental tolerances and interbreeding between morphologically distinct units should result in wide, perhaps ubiquitous distributions^6–8^. On the other hand, current careful investigation of taxonomy and distribution of diatoms^5,9–12^ and other microorganisms^13,14^ have shown that unicellular organisms can be specific to restricted areas. Due to the long history of references that *Hantzschia amphioxys* (Ehrenberg) Grunow has a cosmopolitan distribution^15–17^, an investigation of the global distribution of this species is an important tool for our understanding of global diatom biogeography.

Until recently, the genus *Hantzschia* Grunow included less than 50 species known from different parts of the world and from different water ecosystems like freshwater and saline waterbodies, terrestrial and soil ecosystems^1^. Many new species from this genus were described during the last decade. Over 250 described taxa are currently known from the genus *Hantzschia*^18^. Main morphological features that are used for species delimitation are number of striae and areolae, position and shape of fibulae, the degree and direction of deflection of the external and internal central raphe endings and the shape of the frustule^19^.

The first drawings of valves, which later became the basis for the description of *Hantzschia amphioxys*, were made by C.G. Ehrenberg for several diatoms found in samples from the Americas, including the Falkland Islands, Peru, French Guiana and Labrador Peninsula in 1843; the valve from the Labrador was designated as lectotype^20^. Initially, the species was described as *Eunotia amphioxys*^21^. A. Grunow, discussing new species of diatoms from Honduras, introduced the new genus *Hantzschia*, which included five species previously from the genera *Eunotia* Ehrenberg*, Nitzschia* Hassall and *Epithemia* Kützing^22^.

C.S. Boyer^23^ chose the species *Eunotia amphioxys* as type for the genus *Hantzschia*. The further history of the study of *H. amphioxys* is associated with the accumulation of much material such as distributional reports on this species from around the world, and the inclusion of specimens reported under this name in taxonomic references and floristic studies. In parallel with this, there was an improvement in the methods of algal research, including the application of scanning and transmission electron microscopy, both of which contributed to the identification of new and taxonomically-important features for distinguishing between diatom species. For more than 150 years in the study of *H. amphioxys*, the diagnosis of this species has undergone numerous refinements and revisions. By the end of the first decade of the twenty-first century, many, sometimes completely contradictory, diagnoses of the species were the result. For example, the length and width of valves as in different sources include: 20–100 and 5–15 µm^24^, 20–210 (300) and 13–15 µm^25^, 15–50 and 5–7 µm^19^, 36–68 and 6–10 µm^16^. Such discrepancies in size are also mirrored in the reported number of striae and fibulae in 10 µm. The situation changed in 2014, when R. Jahn, besides designating a lectotype after reviewing the samples of C.G. Ehrenberg, proposed an epitype from a soil strain for *H. amphioxys*^20^.

Until now, the diversity of representatives of the genus *Hantzschia* among soil diatoms was reported as *H. abundans* Lange-Bertalot, *H. amphioxys*, *H. elongata* Grunow and *H. vivax* (W. Smith) Peragallo, including some of their varieties^26–34^. *H. amphioxys*, as the most commonly reported species, has been noted to occur in various ecosystems, from diverse soils, including alkaline^32^ or calcareous^26^, soils of halophilic, shrub, meadow, and psammophytic phytocenoses^30^, soil horizons in artificial and natural forests of the steppe zone^33,35^, forest litter of pine, oak and white acacia plantations^36^, urban soils^37^ and disturbed lands^38^. It was shown that *H. amphioxys* could dominate even in the steppe soil post-pyrogenic communities^39^ and in biological soil crusts after the effects of fire^29^.

At the same time, the adoption of a narrow species concept by many researchers had led to the description of a number of new species within the genus *Hantzschia*. Thus, 17 new taxa were described by Lange-Bertalot et al.^40^ mainly from samples from Sardinia, one taxon from springs in Germany^41^, four species from Xinjiang Province, China^18^, and five new taxa from fresh water bodies and soil of the South Atlantic islands^19^. Until recently, many of the described species had been identified as *H. amphioxys*, which was considered to be a cosmopolitan species.

The predominant use of light microscopy for a long period of time as the main method of studying soil diatoms has led to the conclusion that only a few cosmopolitan species dominate in the soils. However, at the present stage of research, the combination of light and electron scanning microscopy and molecular phylogenetic analyses, can either support cosmopolitanism among soil diatoms or reveal their hidden diversity. Our study presents molecular phylogenetic analyses of 13 novel soil strains of diatom algae, corresponding to *H. amphioxys* in the broad sense, isolated from soil or forest floor in the steppe, forest-steppe, forest and mountain zones of Eurasia. In addition, we performed morphological studies of 12 original strains by Souffreau et al.^16^. The results contain a description of five new species of *Hantzschia* and remarks on the biogeography of *H. amphioxys* sensu stricto.

## Results and discussion

**Figure 1:**
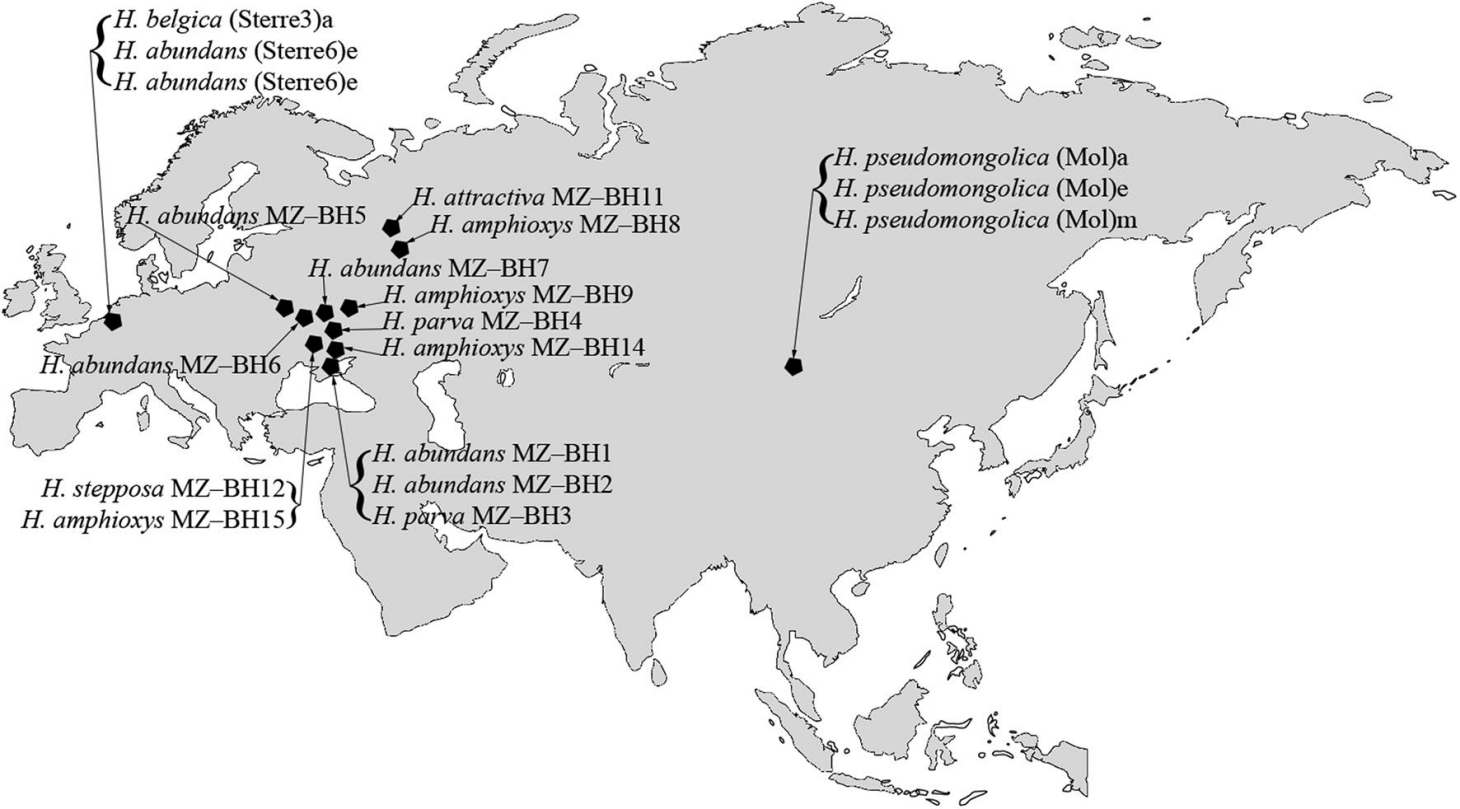
Map with the habitat locations of the studied strains.

In most of the forest soil samples used in this survey, specimens belonging to the genus *Hantzschia* are quite common. Based molecular as well as on light microscopy (LM) and scanning electron microscopy (SEM) observations of 25 strains, seven different taxa were recognized. Figure 1 contains the locations of the strain’s habitats. In anticipation of the nomenclatural consequences, we are using the new names already here but will describe them formally later.

### Molecular data

**Figure 2:**
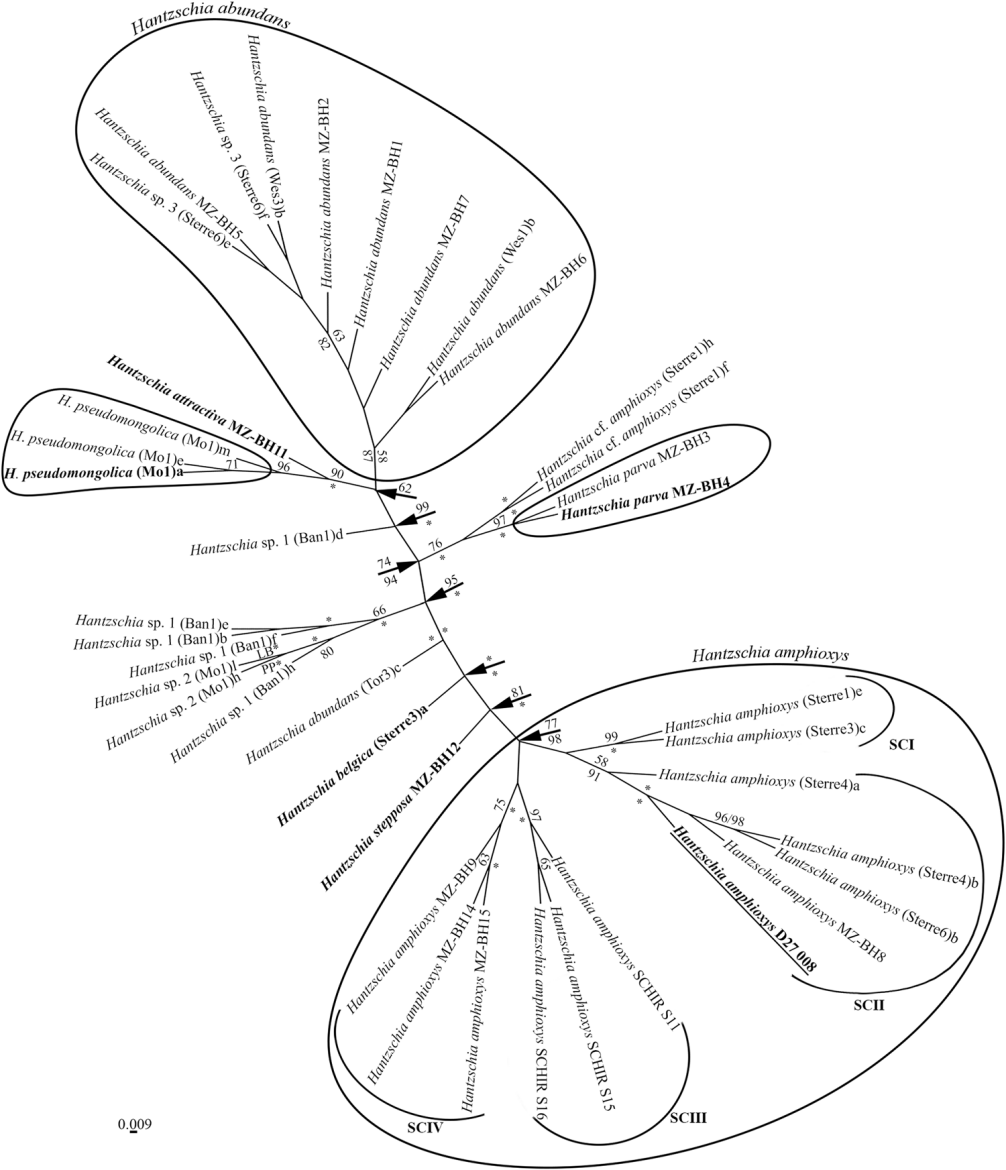
Bayesian tree for representatives of the different strains *Hantzschia*, from an alignment with 40 sequences and 1785 characters (partial *rbc*L gene and 28S rDNA fragments). Type strains indicated in bold. The epitype of *Hantzschia amphioxys* is underlined. Values above the horizontal lines (on the left of slash) are bootstrap support from RAxML analyses (< 50 are not shown); values below the horizontal lines (to the right of slash) are Bayesian posterior probabilities (< 80 are not shown). *100% statistical support.

The obtained phylogenetic tree for representatives of the different strains *Hantzschia* contains several large clades, some of which are monophyletic, while others contain several different species names (Fig. 2). In the analyzed tree, the largest clade is represented by different strains of *H. amphioxys*, the structure of which is described in the corresponding molecular analysis section. At the same time, the most significant is that in the same clade there is strain *H. amphioxys* D27_008, which has been designated as epitype^20^. One of the largest is the clade with *H. abundans*, which, in addition to our strains, and some that have already been published, includes the group of strains referred to as “*Hantzschia* sp. 3” (Sterre6)e, (Sterre6)f from Souffreau et al.^16^. We propose to refer to all of these strains as *H. abundans*. The next clade consists of the new species of *H. attractiva* and three strains of *Hantzschia* sp. 2 (Mo1)a, (Mo1)e, (Mo1)m from Souffreau et al.^16^, the latter we propose to merge into the new species named *H. pseudomongolica*, which is sister to *H. attractiva*. Given the topology of the tree and the morphological features of the representatives, we can conclude that there is a close relation between *H. abundans* and *H. attractiva* plus *H. pseudomongolica*. A separate group consists of two clades with sufficient statistical support (likelihood bootstrap, LB 76; posterior probability, PP 100), one of which is represented by two strains of *H. parva*, and the other with strains of *H.* cf. *amphioxys* (Sterre1)f, (Sterre1)h. Another large clade represents a set of strains of *Hantzschia* sp. 1 and *Hantzschia* sp. 2 (Mo1)h, (Mo1)l from Souffreau et al.^16^, among which there are both large cells (86–89 µm length) and smaller ones (37–39 µm length); strains also differ by striation – from 18–20 striae in 10 μm (strain (Mo1)h) to 21–22 in 10 μm (strain (Ban1)h). It is possible that *Hantzschia* sp. 1 and *Hantzschia* sp. 2 (Mo1)h, (Mo1)l may be several closely related species. Besides the large clades, there are a number of separate branches in the tree, representing separate strains: *Hantzschia* sp. 1 (Ban1)d, and the new species *H. belgica* (*H.* cf. *amphioxys* (Sterre3)a from Souffreaua et al.^16^) and *H. stepposa*. Interesting is the position of the *H. abundans* (Tor3)c strain, which is very distant from other representatives of *H. abundans* and probably is a cryptic taxon, whose taxonomic status needs to be revised.

The absence of nucleotide sequences of the 18S rDNA gene for the *Hantzschia* strains discussed in Souffreau et al.^16^ did not allow us to carry out phylogenetic analysis based on three genes (18S rDNA, 28S rDNA and *rbc*L). However, in GenBank, 18S rDNA sequences are available for other *Hantzschia* strains, including strain D27_008, for which the nucleotide sequence of the 18S V4 rDNA region is known (NCBI Accession FR873247). Comparison of this sequence with similar ones in our strains shows a similarity of 98.4% (368 bp) with *H. amphioxys* MZ–BH9, MZ–BH14 and MZ–BH15. At the same time, the 18S V4 rDNA regions of these three strains are identical and they differ from the epitype by four transitions and one transversion. The similarity with the MZ-BH8 strain is somewhat less with 98.1%, due to one transition (transitions), in which it differs both from the rest of our strains and from the epitype. Also, for the 18S V4 rDNA region of the *H. amphioxys* D27_008 strain, compared with all of our *H. amphioxys* strains, a specific feature is characteristic—the position of one nucleotide (insertion of thymine T). The level of similarity of 98.1–98.4% significantly exceeds the previously calculated rate of 97% for 399/387 bp^20^ between the strain *H*. *amphioxys* D27_008 and the strain *H*. *amphioxys* var. *major* A4 (NCBI Accession HQ912404) from Ruck and Theriot^42^.

The studied *Hantzschia* strains presented low similarities between the clades indicated in the Fig. 2 and the evolutionary distance matrix based on the partial 28S rRNA gene including the D1-D3 region showed that nine *H. abundans* strains shared 99.20–99.87% similarities inside the clade, but they shared 95.61–96.67% similarities with *H. amphioxys* strains, 95.74–96.25% with *H. stepposa* MZ–BH12 and 96.14–96.54% with *H. belgica* (Sterre3)a (Supplementary Table S1). Thirteen *H. amphioxys* strains shared more than 99.07% similarity inside the clade with the greatest differences between SCII and SCIII subclades. Simultaneously, the evolutionary distance matrix showed that *H. pseudomongolica* strains shared low similarities with *Hantzschia* species which have central raphe endings internally bent to opposite directions with similarity 96.01–96.54% for *H. amphioxys*, 96.11% for *H. stepposa* and 96.54% for *H. belgica*.

Pairwise comparisons with putatively related taxa showed that strains of *H. amphioxys* were more than 99.13% similar, however they shared 98.01–98.26% similarities with *H. attractiva* and less than 98.55% amino acids similarity of ribulose-1,5-bisphosphate carboxylase with *H. abundans* strains in the analysis (Supplementary Table S2). Amino acids similarity between *H. belgica*, *H. parva* and *H. stepposa* had value less 99%. The percent similarity among aligned amino acids between *Hantzschia* species had a range of 97.95–100%.

Calculated evolutionary distances for the region D1–D3 of the 28S rRNA gene and amino acid sequence of ribulose-1,5-bisphosphate carboxylase provided strong evidence for *H. attractive, H. belgica, H. parva, H. pseudomongolica* and *H. stepposa* as species separate from *H. amphioxys* and *H. abundans*, since the intraspecies percent dissimilarity for *Hantzschia* species was 0.13–0.93% (D1–D3) or 0.59–0.89% (*rbc*L).

### Morphological comparisons

In order to obtain an overview of the diversity and distribution of the diatoms with *Hantzschia amphioxys*-like morphology, we took soil samples for diatom analysis from steppe, meadow and forest biogeocenoses of Eurasia. In total, we studied 20 soil samples and from these thirteen strains of *Hantzschia* were isolated. *H*. *attractiva* sp. nov., *H*. *belgica* sp. nov., *H*. *parva* sp. nov., *H*. *pseudomongolica* sp. nov. and *H*. *stepposa* sp. nov. were described based on differences in the shape of the valves, significant differences in dimensions, a lower number of striae and areolae in 10 μm and the degree and direction of deflection of the internal central raphe ends. Molecular investigations based on two-gene phylogeny and comparison of 18S V4 rDNA sequences supported our results that new species were clearly separated from other *Hantzschia* species. The remaining species belonged to *H. abundans* and *H. amphioxys*.

**Figure 3:**
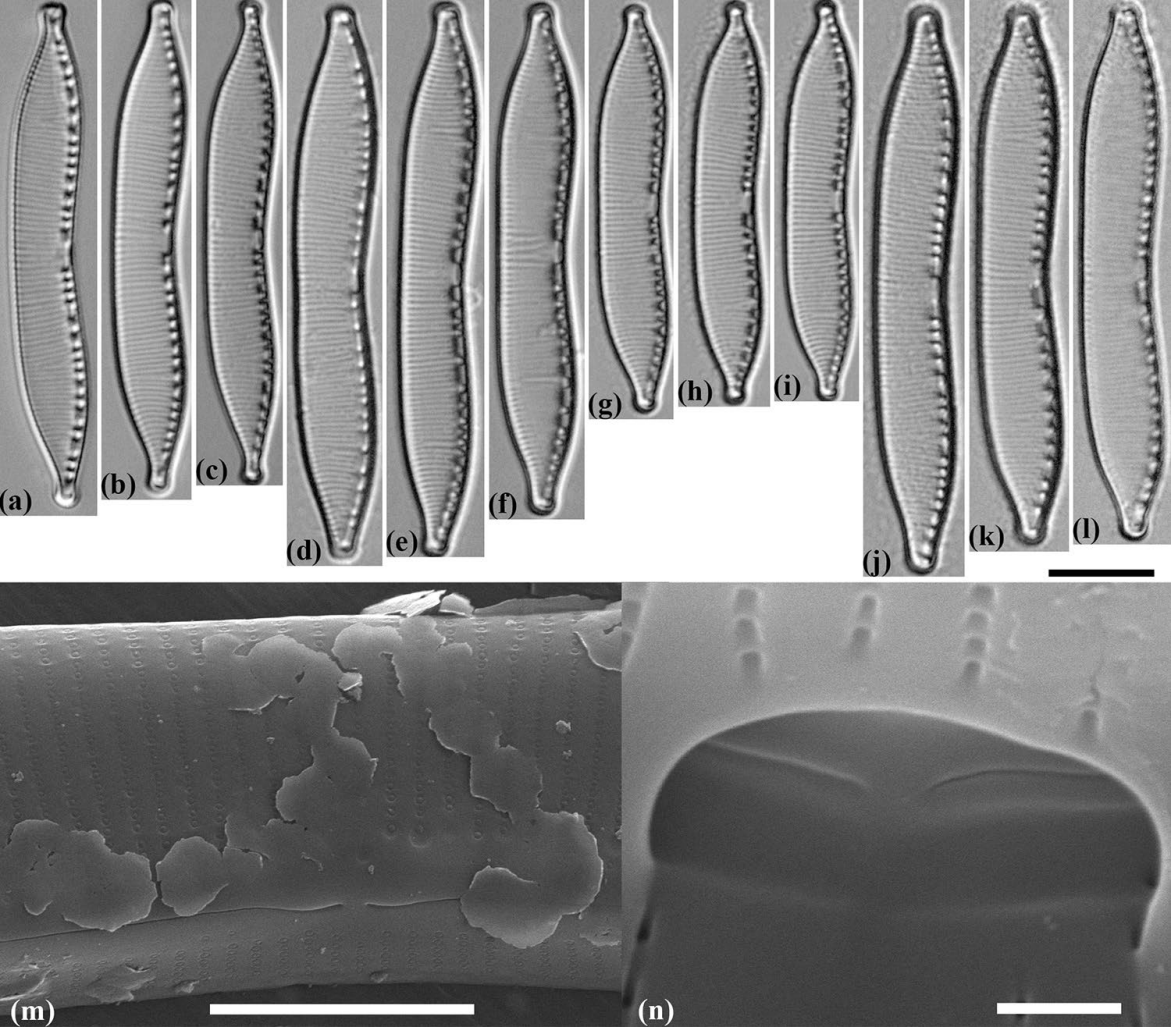
Valve morphology of the different strains of *Hantzschia abundans*. (**a**–**l**) Light micrographs. (**a**–**c**) *H. abundans* MZ–BH1. (**d**–**f**) *H. abundans* MZ–BH2. (**g**–**i**) *H. abundans* MZ–BH6. (**j**–**l**) *H. abundans* MZ–BH7. (**m**) Scanning electron micrograph of *H. abundans* MZ–BH1, exterior of valve at valve center, showing the bent of the raphe near the central raphe endings. (**n**) Scanning electron micrograph of *H. abundans* MZ–BH7, interior of valve at valve center, showing the unilateral bent of the central raphe endings. Scale bars (**a**–**l**) = 10 µm, (**m**) = 5 µm, (**n**) = 0.5 µm.

*H. abundans* was split off from *H. amphioxys* by Lange-Bertalot^28^. The main morphological features of the species (compared to *H. amphioxys*) were the large sizes of the valves (length up to 80 µm, width up to 10 µm), fewer striae in 10 µm (15–20) and one-sided deviation of the internal proximal raphe ends (Table 1). A soil isolate from a wet meadow dominated by *Phalaridetum*, which was located 30 m from the river Nidder, Windecken, Hessen, Germany, was chosen as holotype. In general, our strains correspond to the diagnosis of the *H. abundans* species according to all major morphological characteristics. An exception, however, is the slightly smaller valve length in strain MZ–BH6 (37.5–39 µm, in diagnosis—not less than 40 µm), a smaller valve width in strains MZ–BH1 and MZ–BH6 (6–7 µm, in diagnosis—7–10 µm). We also noted an increase in areolae density up to 50 in 10 µm (40 in 10 µm in diagnosis), although close values (45 in 10 µm) were noted by Zidarova et al.^19^ for the population of *H. abundans* from Byers Peninsula, South Shetland Islands. We have not noted the formation of the keel near the raphe from the outer side of the valve in our strains, which, according to Lange-Bertalot^28^, could indicate an excess of silica in the substrate. From the outer side of the valve in all the studied strains, we noted a slight curvature of the raphe near its central endings, which was not previously noted (Fig. 3m). This feature is more characteristic for different strains of *H. amphioxys*. According to morphological characteristics such as the width and length of the valve, the number of striae and fibulae 10 μm (supplementary Table S2 in Souffreau et al.^16^), as well as the position in the phylogenetic tree *rbc*L–28S rDNA (Fig. 2), we suggest to include the *Hantzschia* sp. 3 strains (Sterre6)e and (Sterre6)f from Souffreau et al.^16^ to *H. abundans*.

**Table 1:** Comparison of species and strains of *Hantzschia* from studied material with similar taxa.

Species, strains	Length (μm)	Width (μm)	Number of striae in 10 μm	Number of fibulae in 10 μm	Number of areolae in 10 μm	Central raphe endings structure	references
*H. abundans* MZ–BH1	45.5–48	6–7	16–20	7–9	40–45	Internally unilaterally bent	this study
*H. abundans* MZ–BH2	48.5–53.5	6.5–7.5	18–20	6–9	40–45	Internally unilaterally bent	this study
*H. abundans* MZ–BH5	48.5–50	6.5–7.5	18–20	7–9	40–50	Internally unilaterally bent	this study
*H. abundans* MZ–BH6	37.5–39	6–6.5	18–20	7–9	45–50	Internally unilaterally bent	this study
*H. abundans* MZ–BH7	50–53.5	7–7.5	16–18	7–8	40–45	Internally unilaterally bent	this study
*H. abundans*	40–80	7–10	15–20	5–8	40	Internally unilaterally bent	Lange-Bertalot^28^
*H. achroma*	12–58	4–4.5	34–35	11–14	n.a	n.a	Li and Volcani^45^
*H. acuticapitata*	57–67	7.8–9.1	22–23	6–9	50	Internally straight	Zidarova et al.^19^
*H. amphioxys* MZ–BH8	36–38	4.8–5	23–24	6–8	45–48	Internally bent to opposite directions	This study
*H. amphioxys* MZ–BH9	40–41.5	5–6	23–24	6–8	40–50	Internally bent to opposite directions	This study
*H. amphioxys* MZ–BH14	44–46	5.5–6	21–24	7–8	50–60	Internally bent to opposite directions	This study
*H. amphioxys* MZ–BH15	40–41.5	5–6	22–23	7–10	45–50	Internally bent to opposite directions	This study
*H. amphioxys* isolectotype	38–42.5	4.4–6.4	23–25	7–11	n.a	n.a	Jahn et al.^20^
*H. amphioxys* epitype	36.8–44.6	5.2–6.6	20–26	5–7.5	48–53	Internally bent to opposite directions	Jahn et al.^20^
*H. attractiva* MZ–BH11	66–70	7.7–8.9	17–18	5–7	40–50	Internally unilaterally bent	this study
*H. bardii*	40–80	6–10	20–24	6–9	36	n.a	Lange-Bertalot et al.^40^
*H. belgica* (Sterre3)a	36–38	6–7	22–23	8–9	50–55	Internally bent to opposite directions	this study; Souffreau et al.^16^
*H. compactoides*	60–80	10–12	14–16	5–6	30	n.a	Lange-Bertalot et al.^40^
*H. delicatula*	43–50	5.5–6	18	7–9	30	n.a	Metzeltin et al.^43^
*H. giessiana*	50–100	6.5–11	16–20	4–7	26–30	Internally bent to opposite directions	Lange-Bertalot^28^
*H. mongolica*	80–127	15–18	11–14	n.a	n.a	n.a	Metzeltin et al.^44^
*H. nematoda*	60–90	8	18	7–9	n.a	n.a	Metzeltin et al.^43^
*H. parva* MZ–BH3	41–42.5	6–7	17–20	7–8	40–45	Internally unilaterally bent	this study
*H. parva* MZ–BH4	37–38.5	5–6	19–20	8–9	40–45	Internally unilaterally bent	This study
*H. psammicola*	50–68	8	10	n.a	28	Internally bent to opposite directions	Metzeltin et al.^43^
*H. pseudomongolica*	83.2–91.4	7.8–9.4	19–21	6–8	40–45	Internally straight	This study; souffreau et al.^16^
*H. stepposa* MZ–BH12	40–42.5	6–6.5	22–25	5–9	44–52	Internally bent to opposite directions	This study
*H. subrupestris*	50–80	7–9	14–16	5–7	24	Internally straight	Lange-Bertalot^28^
*H. valdeventricosa*	(41) 55–70	9–10	18–19 (20)	5.5–7	n.a	n.a	Metzeltin et al.^43^

*H. attractiva* sp. nov. is visually and parametrically similar with some *Hantzschia* species but differs in a number of ways. In terms of the ratio of the length and width of the valve, the shape of its ends and concavity in the center of the ventral side of the valve, *H. attractiva* is most similar to *H. subrupestris* Lange-Bertalot^28^. However, these species can be distinguished from one another by a smaller number of striae (14–16 in 10 μm) and areolae (24 in 10 μm) in *H. subrupestris* (Table 1). In the case of *H. subrupestris*, the central raphe ends are straight or barely noticeably deflected in one direction, whereas in *H. attractiva* they deviate in one direction very clearly. Compared with *H. abundans*, the diagnostic features of *H. attractiva* include: less pronounced concavity of the middle part of the valve from the ventral side, vertical position of the valve ends, which are also less pronounced; a larger number of areolae in 10 μm and one-sided deviation of the central raphe ends on both sides of the valve (they are located straight in *H. abundans*). Our species differs from *H. bardii* Lange-Bertalot, Cavacini, Tagliaventi et Alfinito in the shape of the valve ends (in *H. bardii*, they are obliquely cuneate and finally protracted narrow-rostrate to subcapitate), a smaller number of striae in 10 µm and a larger number of areolae^40^. Of nearly similar size to *H. attractiva* is *H. compactoides* Lange-Bertalot, Cavacini, Tagliaventi et Alfinito, which can be distinguished by a more pronounced bend of the valve from the ventral side, the direction of the valve ends in the same direction and their rostrate shape^40^. Also, *H. attractiva* is characterized by smaller values of the width of the valve, the greater the number of striae and areolae in 10 µm. Another similar species is *H. giessiana* Lange-Bertalot et Rumrich^28^, which characterized by capitate valve ends, a smaller number of areolae in 10 µm (26–30) and the central raphe ends from inner part deviate in opposite directions in comparison with *H. attractiva*. The lengths 66–70 µm and breadth 7.7–8.9 µm also correspond to the type of *H. nematoda* Metzeltin, Lange-Bertalot et García-Rodríguez and *H. psammicola* Garcia-Baptista, which are distinguished from *H. attractiva* in addition to the shape of the valves by the presence of costae on the outer side of the valve^43^.

We obtained light and scanning electron micrographs based on the study of the original materials of the strains (Mo1)a, (Mo1)e and (Mo1)m and described *H. pseudomongolica* sp. nov. Originally the (Mo1)a, (Mo1)e and (Mo1)m strains were presented in Souffreau et al.^16^ as “*H. amphioxys* sp. 2”, however, the authors presented only the length and breadth characteristics of the valves without clarifying the taxonomic status or providing an explanation of the isolated position of the strains in the phylogenetic tree in relation to the clade with *H. amphioxys* (Fig. 4 in Souffreau et al.^16^). *H. mongolica* Metzeltin, Lange-Bertalot et Nergui ^44^ was described from freshwater habitats from Mongolia. This species differs from *H. pseudomongolica* sp. nov. by its larger valve size (length 80–127 µm and width—15–18 µm) and significantly smaller striae density (11–14 in 10 µm). *H. pseudomongolica* sp. nov. differs from *H. attractiva* in both its dimensional characteristics (longer valves and more dense arrangement of striae) and the shape of the valve ends. In *H. attractiva* the ends are narrowed, broadly-rounded and directed vertically, in *H. pseudomongolica* the ends are more attenuate, almost capitate, and deflected towards to the dorsal side.

The paratype of *H. parva* differs from the holotype by somewhat larger valve sizes, namely, 41.5–42.5 µm long and 6–7 µm wide. The large width of the valves, a smaller number of striae in 10 µm and one-sided deviation of the central ends of the raphe from the inside of the valve in *H. parva* underlines its differences with *H. amphioxys* (in comparison with the isolectotype^20^). Compared with *H. abundans, H. parva* is characterized by having narrower valves (for *H. abundans* in the range of 7–10 µm), a more dense areola arrangement, lack of a keel near the raphe externally and by the position of the central raphe ends (for *H. abundans* they are straight internally).

The smallest species of the genus *Hantzschia* is *H. achroma* B.E. Volcani et C.-W. Li, which differs from *H. parva*, in addition to the narrower shape of the valve and tapering valve ends, by a smaller width (up to 4.5 μm), more dense striation (34–35 striae in 10 μm) and a higher fibular density (11–14 in 10 μm)^45^. In spite of the close dimensions of the valve, described from Uruguay *H. delicatula* Metzeltin, Lange-Bertalot et García-Rodríguez^43^ has an almost flat ventral side of the valve and its ends are in almost the same plane as the cuneate, as well as lower density of areolae (30 to 10 μm), which distinguishes it from *H. parva* (Table 1). The greater width of the valve (9–10 µm), as well as its flattened form on the ventral side, distinguish *H. parva* from another Uruguayan species *H. valdeventricosa* Metzeltin, Lange-Bertalot et García-Rodríguez^43^.

According to its morphometric features, *H. stepposa* sp. nov. is similar to *H. amphioxys*. Also, these two species are similar in the deflection of the central raphe ends on the inner side of valve—they curve in opposite directions. Despite this, the two species are easily distinguished by the shape of the valves: *H. amphioxys* is characterized by a pronounced concavity of the ventral side of the valve in the middle, whereas in *H. stepposa*, the ventral side is almost straight; in *H. amphioxys*, the valve ends are clearly directed to the dorsal side of the valve, while in *H. stepposa* they are arranged vertically. The presence of central raphe ends at the inner side that are deflected in opposite directions distinguishes *H. stepposa* from most of the known *Hantzschia* species, whose valve length do not exceed 100 μm (including *H. attractiva* and *H. parva*). The only species with a similar position of the central ends of the raphe and up to 100 µm in length is *H. giessiana*^28^. However, along with the large size of the valve (50–100 μm), it is also characterized by a completely different shape of the valves—a strongly concave ventral side and ends curved towards it (Table 1).

Originally strain (Sterre3)a was presented in Souffreau et al.^16^ as “*H.* cf. *amphioxys*”, however, the authors presented only the morphometric characteristics and the light micrograph of one valve without clarifying its taxonomic status and explaining its isolated position in the phylogenetic tree in relation to the *Hantzschia amphioxys* clade (Fig. 4 in Souffreau et al.^16^). We present light and scanning electron micrographs based on a study of the original material with description of a new species *H. belgica* sp. nov. In comparison with other *Hantzschia* species that have internal proximal raphe ends that are deflected in opposite direction (*H. amphioxys* and *H. stepposa*), *H. belgica* differs in shape and direction of the valve ends. If *H. amphioxys* and *H. stepposa* are characterized by extended and capitated ends, which are directed to the dorsal side of the valve in *H. amphioxys* and arranged vertically in *H. stepposa*, in *H. belgica* the ends of the valves are not distinctly protracted and not directed to either the dorsal or ventral margins.

A comparative analysis of the main morphological characteristics (length and width of the valves, the number of striae, the number of fibulae and areolae in 10 μm) in the four *H. amphioxys* strains isolated by us with the proposed diagnoses of Jahn et al.^20^ of the lectotype and epitype show a coincidence in most data. Small differences can be attributed to the slightly larger maximum length of the valves in strain MZ–BH14 (46.0 μm with a maximum of 44.6 μm in the epitype). The density of the areolae for epitype is in the range of 48–53 areolae in 10 µm (submitted in this study). Some studies indicate the number of areolae in 10 µm for different populations of *H. amphioxys* in the range of 40–50 areolae in 10 µm^19,28^. In this regard, the excess of this range is observed only in the strain MZ–BH14, whose density of areolae in the valve can reach 60 in 10 µm.

Based on a detailed study of morphology and ultrastructure, as well as the use of phylogenetic analysis using the 28S rDNA and *rbc*L genes, our results found 25 soil strains that represent geographically distant populations and formally could be classified as *H. amphioxys* sensu lato, containing seven different *Hantzschia* species, including five new for science. Five strains were identified as *H. abundans* and are fully consistent with the diagnosis of all major morphological characteristics for that taxon. For these strains, we noted areolar densities up to 50 in 10 μm (the original diagnosis reported 40 areolae in 10 μm). Externally, in all the studied strains, we noted a slight curvature of the raphe near its proximal endings, which was not previously reported. In addition, another four of the studied strains represent different populations of *H. amphioxys* and their morphological characteristics fully correspond to the adopted isolectotype and epitype^20^. The main specific features of these types include: the presence of 21–25 striae in 10 µm, 6–11 fibula in 10 µm, 40–50 areolae in 10 µm and internal proximal raphe ends curved in opposite directions (Table 1). Five new species were identified on the basis of differences in the shape of valves, the size characteristics of the valves, the number of striae and areolae in 10 μm and the position of the central raphe ends compared to the already known *Hantzschia* species.

Phylogenetic analysis using the 28S rDNA and *rbc*L genes showed that adding our strains does not change the tree topology obtained by C. Souffreaua et al.^16^ for different lines of *H. amphioxys*. At the same time, the presence of a detailed morphological description of our strains using SEM results made it possible to distinguish morphological differences between the available clades. The connection of the topology of the obtained tree with the morphological features of the isolated strains shows that morphological features reflect phylogenetic relatedness. These morphological features that also reflect relationships based on genomic data are shape of the valve, the raphe structure (presence or absence of the central nodule and direction of the central branches relative to each other in it), the number of striae and areolae in 10 µm. These morphological features were correlated with the clades in the ML (Maximum Likelihood) and BI (Bayesian inference) trees constructed from fragments of the 28S rDNA and *rbc*L genes.

### Description of new species and emendations

#### Hantzschia abundans Lange-Bertalot emended by Maltsev et Kulikovskiy (Fig. 3)

##### Morphology

Valves are dorsiventral with evidently concave central part of ventral side of the valve. Dorsal side is slightly convex, sometimes straight (Fig. 3a–l). Valve ends are slightly tapered and capitate (strains MZ–BH2, MZ–BH5) or more cuneate and capitate (MZ–BH1, MZ–BH6). Length 37.5–53.5 µm, breadth 6.0–7.5 µm. Fibulae are of different sizes, 6–9 in 10 µm. Two central fibulae are rather widely spaced. Striae are radiate, parallel at the valve ends, 16–20 in 10 µm. Areolae are not visible in LM. Fibulae are connected with 1–4 striae or interstriae.

##### Ultrastructure

In outside view central raphe ends are straight or slightly curved to the same side (Fig. 3m). In inside view central raphe ends are curved to dorsal side (Fig. 3n). Striae are uniseriate, 40–50 in 10 µm. Helictoglossae are small.

##### Molecular analysis

In the phylogenetic tree based on the 28S rDNA and *rbc*L genes, *H. abundans* strains form a separate clade (Fig. 2). In addition to our 5 strains, we also included two other strains of *H. abundans*, (Wes1)b and (Wes3)b, that were isolated from De Panne, Belgium (Souffreau et al.^16^). This clade also includes soil strains (Sterre6)e and (Sterre6)f, named by Souffreau et al.^16^ as “*Hantzschia* sp. 3”, isolated from Gent, Belgium.

##### Ecology and distribution

The strains *H. abundans* MZ–BH1, MZ–BH2, MZ–BH5, MZ–BH6 and MZ–BH7 isolated by us belong to soil populations (Fig. 1, Table 2).

#### Hantzschia attractiva Maltsev et Kulikovskiy sp. nov. (Fig. 4)

##### Holotype

**Figure 4:**
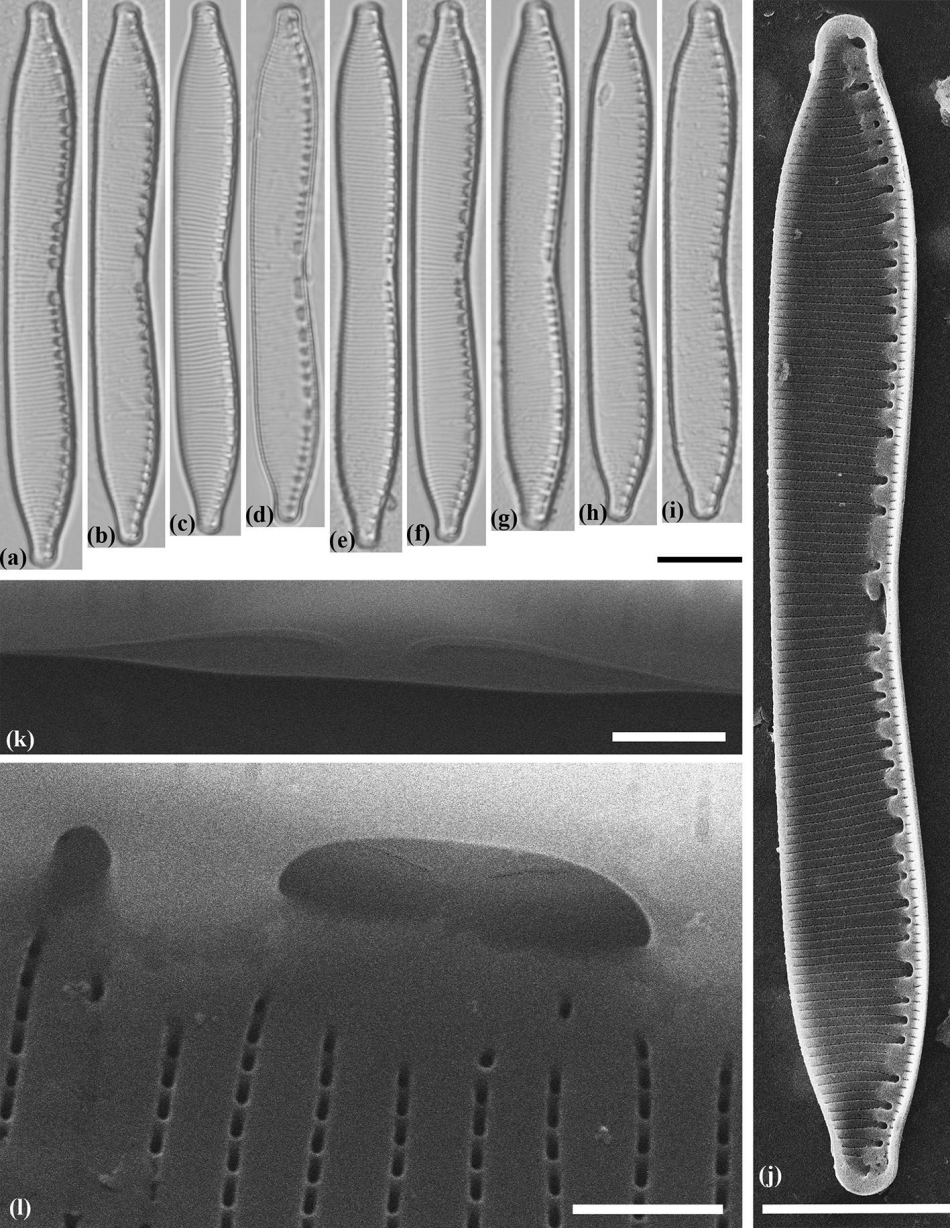
Valve morphology of the strain *Hantzschia attractiva* MZ–BH11. (**a**–**d**) Light micrographs of the natural population. (**e**–**i**) Light micrographs of monoculture. (**j**) Scanning electron micrographs, interior of valve. (**k**) Scanning electron micrograph, showing the raphe keel. (**l**) Scanning electron micrograph, interior of valve at valve center, showing the bent of the raphe near the central raphe endings. Scale bars (**a**–**j**) = 10 µm, (**k**–**l**) = 1 µm.

Slide no. 02508/MZ–BH11 (Holotype represented by Fig. 4) in the collection of Maxim Kulikovskiy, К.A. Timiryazev Institute of Plant Physiology RAS, 10.05.2015, collected by Y. Maltsev.

##### Type locality

Sod-medium podzolic soil, horizon 0–5 cm, plantation *Betula pendula* Roth, Yaroslavl region, Russia, N57°50′ 23.83″ E38°4′13.02″, 10.05.2015.

##### Registration

https://phycobank.org/102516.

##### Diagnosis

Length 66–70 µm, breadth 7.7–8.9 µm. Valves slightly asymmetrical to the apical axis, with dorsal margin concave or straight, ventral margin slightly concave. Apices protracted, rounded. Fibulae are different in size and placed irregularly, 5–7 in 10 µm. Central fibulae are more widely spaced than the others. Striae 17–18 in 10 µm. Areolae are not evident in LM.

##### Ultrastructure

Raphe with two branches, not continuous, internal proximal raphe ends curved in the dorsal direction. Fibulae of varying sizes, connected with 1–5 striae. Striae are uniseriate with 40–50 areolae in 10 µm. Internally, the areolae are rectangular in shape.

##### Etymology

The epithet refers to the attractive nature of the shape of the valve.

##### Molecular analysis

In the phylogenetic tree based on the 28S rDNA and *rbc*L genes, the strain *H. attractiva* MZ–BH11 occupies an isolated position in the clade with three strains of *Hantzschia* sp. 2 (Mo1)a, (Mo1)e and (Mo1)m (Fig. 2), described by Souffreau et al.^16^ from Kangai-Nuruu (Mongolia). Clade *H. attractiva* plus *H. pseudomongolica* (*Hantzschia* sp. 2(Mo1)a, (Mo1)e and (Mo1)m) has high BI support and is sister to the clade of *H. abundans* (Fig. 2).

##### Ecology and distribution

The strain *H. attractiva* MZ–BH11 was isolated from the soil (sod-medium podzol) (Table 2).

**Table 2:** List of all strains examined in this study with their GenBank accession numbers (BOLD ID). Geographic position and description of sample sites indicated.

Strains	Sample locality	Collection date	Latitude	Longitude	Short description	Slide No	GenBank Accession Numbers (BOLD ID)			
							SSU rDNA, partial	LSU rDNA, partial		*rbc*L, partial
*Hantzschia abundans* MZ–BH1	Henichesk, Kherson region, Ukraine	01 Mar 2013	N46°10′31.0″	E34°46′12.1″	Steppe, chestnut soil, horizon 0–5 cm	02502	MW396843	MW396827		MW387960
*Hantzschia abundans* MZ–BH2	Henichesk, Kherson region, Ukraine	01 Mar 2013	N46°9′57.72″	E34°47′34.78″	Cemetery, chestnut soil, horizon 0–5 cm	02503	MW396844	MW396828		MW387961
*Hantzschia abundans* MZ–BH5	Kalinovka village, Zhytomyr region, Ukraine	18 Apr 2015	N50°16′4.03″	E28°47′54.57″	*Betula pendula* forest, chernozem, horizon 0–5 cm	02510	MW396847	MW396831		MW387964
*Hantzschia abundans* MZ–BH6	Dmitrovka village, Kyiv region, Ukraine	18 Apr 2015	N50°26′49.26″	E30°11′8.13″	*Pinus sylvestris* forest, chernozem, horizon 0–5 cm	02511	MW396848	MW396832		MW387965
*Hantzschia abundans* MZ–BH7	Boryspil, Kyiv region, Ukraine	19 Apr 2015	N50°19′54.99″	E31°0′0.50″	*Quercus robur* L. forest, chernozem, horizon 0–5 cm	02512	MW396849	MW396833		MW387966
*Hantzschia abundans* (Sterre6)e	Gent, Oost-Vlaanderen (East Flanders), Belgium	21 Nov 2007	N51°01′30.8″	E3°42′55.2″	Concrete pavement with biofilm, moist	CSO92	n.a		(HANT021-11)	
*Hantzschia abundans* (Sterre6)f	Gent, Oost-Vlaanderen (East Flanders), Belgium	21 Nov 2007	N51°01′30.8″	E3°42′55.2″	Concrete pavement with biofilm, moist	CSO93	n.a		(HANT022-11)	
*Hantzschia attractiva* MZ–BH11	Yaroslavl region, Russia	10 May 2015	N57°50′23.83″	E38°4′13.02″	*Betula pendula* and *Pinus sylvestris* forest, sod-medium podzolic soil, horizon 0–5 cm	02508	MW396852	MW396836		MW387969
*Hantzschia pseudomongolica* (Mo1)a	Kangai-Nuruu, Mongolia	n.a	N47°50′20.0″	E99°59′59.9″	Sediment (littoral zone)	CSO77	n.a		(HANT006-11)	
*Hantzschia pseudomongolica* (Mo1)e	Kangai-Nuruu, Mongolia	n.a	N47°50′20.0″	E99°59′59.9″	Sediment (littoral zone)	CSO78	n.a		(HANT007-11)	
*Hantzschia pseudomongolica* (Mo1)m	Kangai-Nuruu, Mongolia	n.a	N47°50′20.0″	E99°59′59.9″	Sediment (littoral zone)	CSO81	n.a		(HANT010-11)	
*Hantzschia parva* MZ–BH3	Henichesk, Kherson region, Ukraine	01 Mar 2013	N46°9′57.72″	E34°47′34.78″	Cemetery, chestnut soil, horizon 5–10 cm	02504	MW396845	MW396829		MW387962
*Hantzschia parva* MZ–BH4	Kobilyaky village, Poltava region, Ukraine	19 Apr 2015	N49°8′31.69″	E34°13′1.51″	*Quercus robur* and *Fraxinus excelsior* L. forest, chernozem, horizon 0–5 cm	02509	MW396846	MW396830		MW387963
*Hantzschia stepposa* MZ–BH12	Kamenka-Dniprovska, Zaporizhia region, Ukraine	20 Oct 2014	N47°30′23.46″	E34°23′15.99″	Planting of *Quercus robur*, fermentation sub-horizon of forest floor	02516	MW396853	MW396837		MW387970
*Hantzschia belgica* (Sterre3)a	Gent, Oost-Vlaanderen (East Flanders), Belgium	21 Nov 2007	N51°01′38.6″	E3°42′58.4″	The upper half cm of bare soil under *Quercus* in meadow, moist	CSO86	n.a		(HANT015-11)	
*Hantzschia amphioxys* MZ–BH8	Mishutino village, Moscow region, Russia	05 Oct 2015	N56°22′55.12″	E38°6′36.13″	*Populus nigra* L. and *Betula pendula* forest, sod-medium podzolic soil, horizon 0–5 cm	02505	MW396850	MW396834		MW387967
*Hantzschia amphioxys* MZ–BH9	Kursk region, Russia	02 May 2015	N51°41′8.11″	E36°3′57.79″	*Quercus robur* forest, gray forest soil, horizon 0–5 cm	02506	MW396851	MW396835		MW387968
*Hantzschia amphioxys* MZ–BH14	Bogatyr village, Zaporizhia region, Ukraine	04 May 2015	N46°37′8.87″	E35°16′27.13″	planting of *Pinus pallasiana*, fermentation sub-horizon of forest floor	02513	MW396854	MW396838		MW387971
*Hantzschia amphioxys* MZ–BH15	Kamenka-Dniprovska, Zaporizhia region, Ukraine	07 Oct 2014	N47°30′32.50″	E34°23′17.87″	Planting of *Pinus sylvestris*, chernozem with low humus, horizon 0–5 cm	02514	MW396855	MW396839		MW387972

#### Hantzschia pseudomongolica Maltsev et Kulikovskiy sp. nov. (Fig. 5)

**Figure 5:**
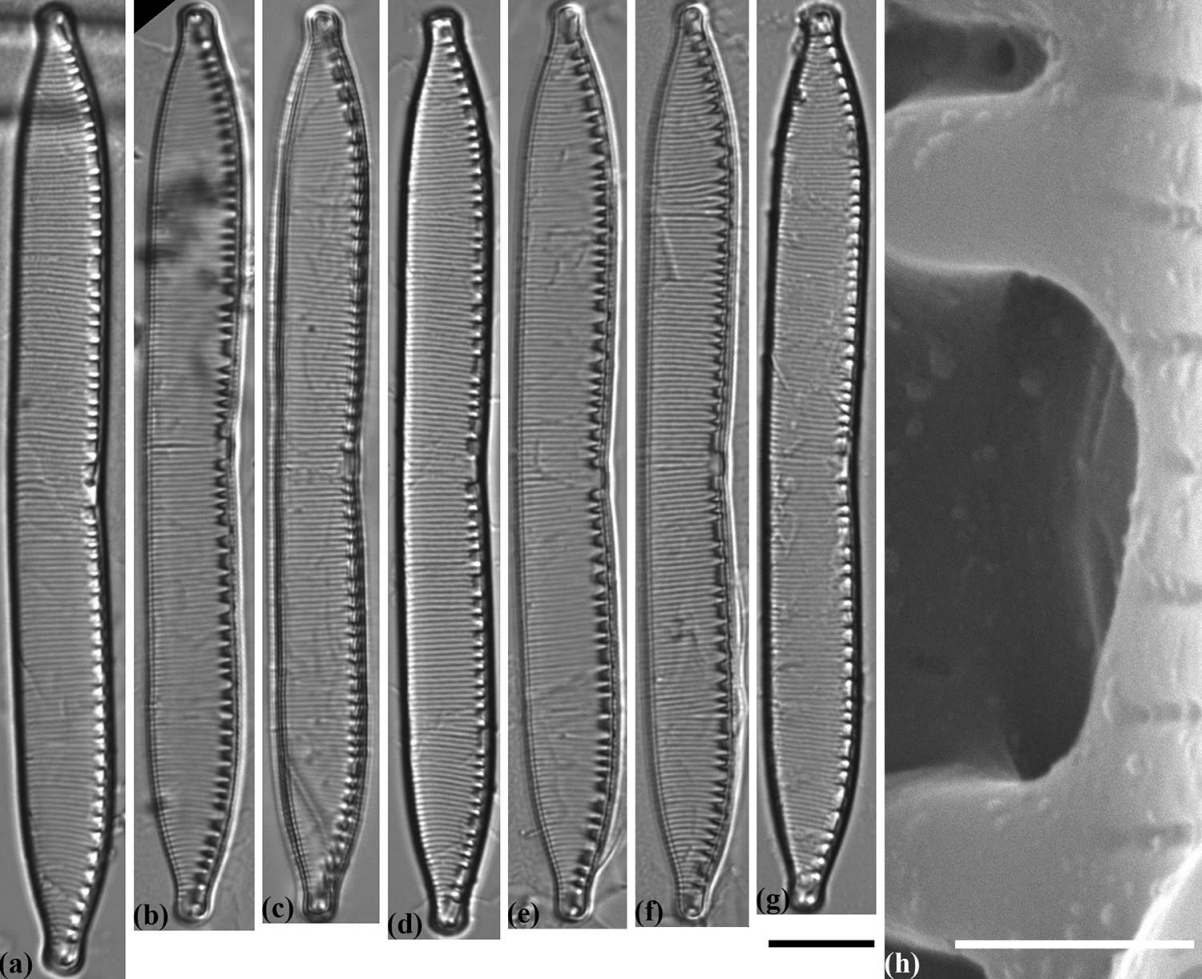
Valve morphology of the strain *Hantzschia pseudomongolica* (Mo1)a. (**a**–**c**) Light micrographs of the natural population. (**d**–**g**) Light micrographs of monoculture. (**h**) Scanning electron micrograph, interior of valve at valve center, showing the position of the central raphe endings. Scale bars (**a**–**g**) = 10 µm, (**h**) = 1 µm.

##### Holotype

Slide no. CSO77/(Mo1)a (holotype represented by Fig. 5a, d–g) in the collection of Universiteit Gent, collected by Pieter Vanormelingen.

##### Type locality

Soil, Kangai-Nuruu, Mongolia. N47°50′20.0″ E99°59′59.9″.

##### Paratype

Slide no. CSO78/(Mo1)e (paratype represented by Fig. 5b–c) in the collection of Universiteit Gent, collected by Pieter Vanormelingen. Slide no. CSO81/(Mo1)m in the collection of Universiteit Gent, collected by Pieter Vanormelingen.

##### Registration

https://phycobank.org/102517.

##### Diagnosis

Valves nearly straight, slightly asymmetrical to the apical axis and some specimens are also slightly asymmetrical to the transapical axis (Fig. 5d,g). Apices protracted, rounded to slightly capitate. Length 83.2–91.4 µm, breadth 7.8–9.4 µm. Fibulae are of different sizes, 6–8 in 10 µm. Two central fibulae are more widely spaced than the others. Striae 19–21 in 10 µm. Areolae are not discernible in LM.

##### Ultrastructure

Raphe not continuous. In inside view central raphe ends are straight (Fig. 5h). Areolae 40–45 in 10 µm.

##### Molecular analysis

In the phylogenetic tree based on the 28S rDNA and *rbc*L genes, *H. pseudomongolica* strains form a common clade with *H. attractiva* with high statistical support (LB 90; PP 100) (Fig. 2). In this case, the strains (Mo1)a and (Mo1)e form a separate subclade, slightly isolated from the clade with strain (Mo1)m.

##### Etymology

The species is named according to the region where it was found and indicating superficial similarities with *H. mongolica*.

#### Hantzschia parva Maltsev et Kulikovskiy sp. nov. (Fig. 6)

**Figure 6:**
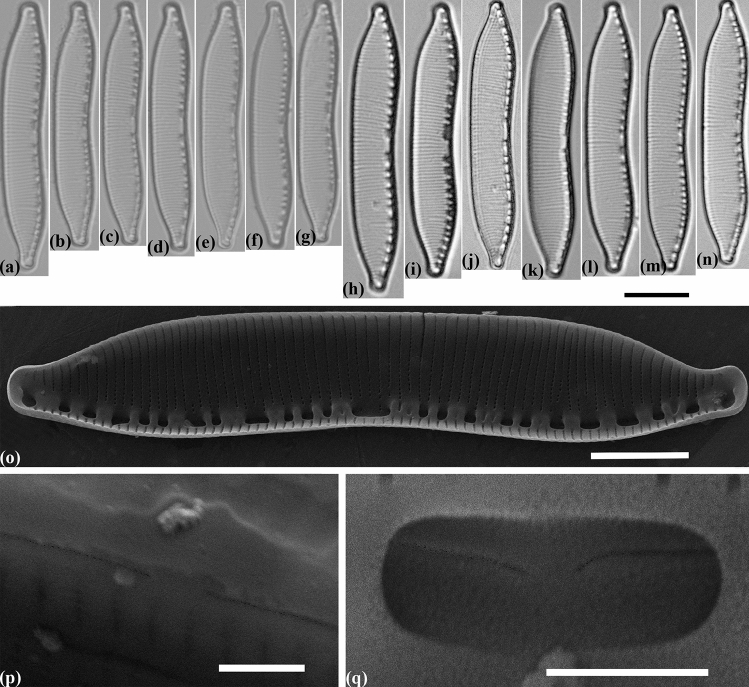
Valve morphology of representatives from the different strains *Hantzschia parva*. (**a**–**n**) Light micrographs. (**a**–**c**) Natural population of *H. parva* MZ–BH4. (**d**–**g**) Monoculture of *H. parva* MZ–BH4. (**h**–**j**) Natural population of *H. parva* MZ–BH3. (**k**–**n**) Monoculture of *H. parva* MZ–BH3. (**o**) Scanning electron micrographs of *H. parva* MZ–BH4, interior of valve. (**p**) Scanning electron micrograph of *H. parva* MZ–BH4, exterior of valve at valve center, showing almost straight the central raphe endings. (**q**) Scanning electron micrograph of *H. parva* MZ–BH3, interior of valve at valve center, showing the unilateral bent of the central raphe endings. Scale bars (**a**–**n**) = 10 µm, (**o**) = 5 µm, (**p**,**q**) = 1 µm.

##### Holotype

Slide no. 02509/MZ–BH4 (Holotype here represented by Fig. 6a–g) in the collection of Maxim Kulikovskiy, К.A. Timiryazev Institute of Plant Physiology RAS, 19.04.2015, collected by Y. Maltsev.

##### Type locality

Chernozem, horizon 0–5 cm, mixed deciduous stands, Poltava region, Ukraine, N49°8′31.69″ E34°13′1.51″, 19.04.2015.

##### Paratype

Slide no. 02504/MZ–BH3 (Paratype here represented by Fig. 6h–n) in the collection of Maxim Kulikovskiy, К.A. Timiryazev Institute of Plant Physiology RAS, 1.03.2013, collected by Y. Maltsev.

##### Registration

https://phycobank.org/102518.

##### Diagnosis

Valves linear-lanceolate, asymmetrical about the apical axis, with dorsal margin more or less straight to slightly convex, ventral margin concave, with apices small, protracted, rounded to subrostrate. Length 37.0–42.5 µm, breadth 5–7 µm. Fibulae irregularly-placed along the ventral margin, 7–9 in 10 µm, with a distinct central nodule. Striae 17–20 in 10 µm; areolae not visible with LM.

##### Ultrastructure

Raphe not continuous, with internal proximal raphe ends deflected slightly in the same direction. Areolae 40–45 in 10 µm, rectangular in shape internally.

##### Etymology

The epithet “*parva*” chosen according to size of the valve and compared with most species in the genus.

##### Molecular analysis

In the phylogenetic tree based on the 28S rDNA and *rbc*L genes, two strains of *H. parva*, MZ–BH3 and MZ–BH4, form a single clade (Fig. 2). With high bootstrap support, a sister clade joins this lineage, which includes two soil strains of *H.* cf. *amphioxys* (Sterre1)f and (Sterre1)h, isolated from the region of Gent, Belgium^16^. Compared to *H. parva*, these strains are characterized by somewhat larger valve sizes (35–49 µm long, 8–9 µm wide) and the number of striae is about 20–22 in 10 µm. It is possible that strains (Sterre1)f and (Sterre1)h represent another new species of *Hantzschia*, closely related to *H. parva*.

##### Ecology and distribution

In the course of this research, two *H. parva* soil strains were isolated from geographically distant biogeocenoses (Fig. 1, Table 2). The strain *H. parva* MZ–BH4 (holotype) was isolated from the soil in the broadleaf forest biogeocenosis. The strain *H. parva* MZ–BH3 (paratype) BH2 was isolated from the soil of the steppe biogeocenosis at the city cemetery. The dominant grass cover consisted of *Elytrigia repens* (L.) Desv. ex Nevski, *Hordeum leporinum* Link and other grasses.

#### Hantzschia stepposa Maltsev et Kulikovskiy sp. nov. (Fig. 7)

**Figure 7:**
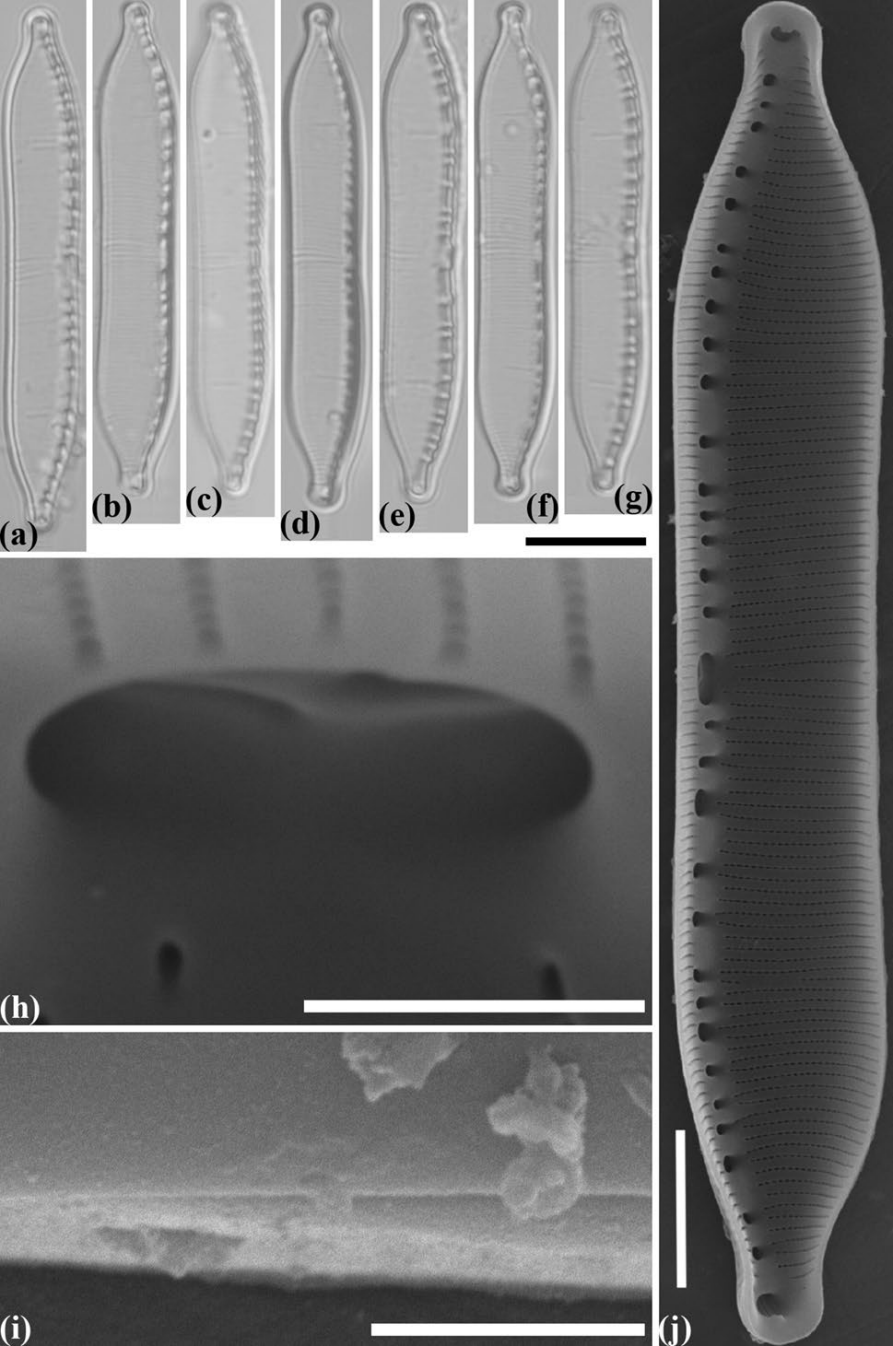
Valve morphology of the strain *Hantzschia stepposa* MZ–BH12. (**a**–**g**) Light micrographs. (**h**) Scanning electron micrograph, interior of valve at valve center, showing bent to opposite directions of the central raphe endings. (**i**) Scanning electron micrograph, exterior of valve at valve center, showing almost straight the central raphe endings. (**j**) Scanning electron micrograph, interior of valve. Scale bars (**a**–**g**) = 10 µm, (**h**,**i**) = 1 µm, (**j**) = 5 µm.

##### Holotype

Slide no. 02516/MZ–BH12 (Holotype represented by Fig. 7) in the collection of Maxim Kulikovskiy, К.A. Timiryazev Institute of Plant Physiology RAS, 20.10.2014, collected by Y. Maltsev.

##### Type locality

Forest litter, ordinary humus chernozem, plantation *Quercus robur*, Zaporizhzhia region, Ukraine, N47°30′23.46″ E34°23′15.99′, 10.20.2014.

##### Registration

https://phycobank.org/102519.

##### Diagnosis

Valves linear-lanceolate, straight to extremely weakly asymmetrical to the apical axis, apices protracted, knob-like to nearly capitate. Length 40.0–42.5 µm, breadth 6.0–6.5 µm. Fibulae irregular in size, 5–9 in 10 µm. Striae 22–25 in 10 µm.

##### Ultrastructure

Raphe not continuous externally and internally; central raphe endings are straight in outside view and curved in opposite directions in inside view. Areolae fine, 44–52 in 10 µm, rectangular in shape internally.

##### Etymology

The species named according to the ecological region where it was found.

##### Molecular analysis

In the phylogenetic tree based on the 28S rDNA and *rbc*L genes, *H. stepposa* MZ–BH12 occupies a separate position (Fig. 2). *H. stepposa* is the sister taxon to the *H. amphioxys* clade, which emphasizes the close relation of the two species. The *H. stepposa* plus *H. amphioxys* clade is sister to the soil-dwelling *Hantzschia belgica* (Sterre3)a, characterized as a small cell species (length 36–37 μm and width 6.0–6.5 μm) with a number of striae close to *H. stepposa* (22–23 in 10 μm).

##### Ecology and distribution

The strain *H. stepposa* MZ–BH12 was isolated from the fermentation sub-horizon of the forest floor in the *Quercus robur* plantation with *Celtis occidentalis* L. in the second tier. The grass ground cover was represented by *Stellaria holostea* L., *Anthriscus sylvestris* (L.) Hoffm., *Glechoma hederacea* L. and *Urtica dioica* L.

#### Hantzschia belgica Maltsev et Kulikovskiy sp. nov. (Fig. 8; Fig. 1 L in Souffreau et al^16^)

**Figure 8:**
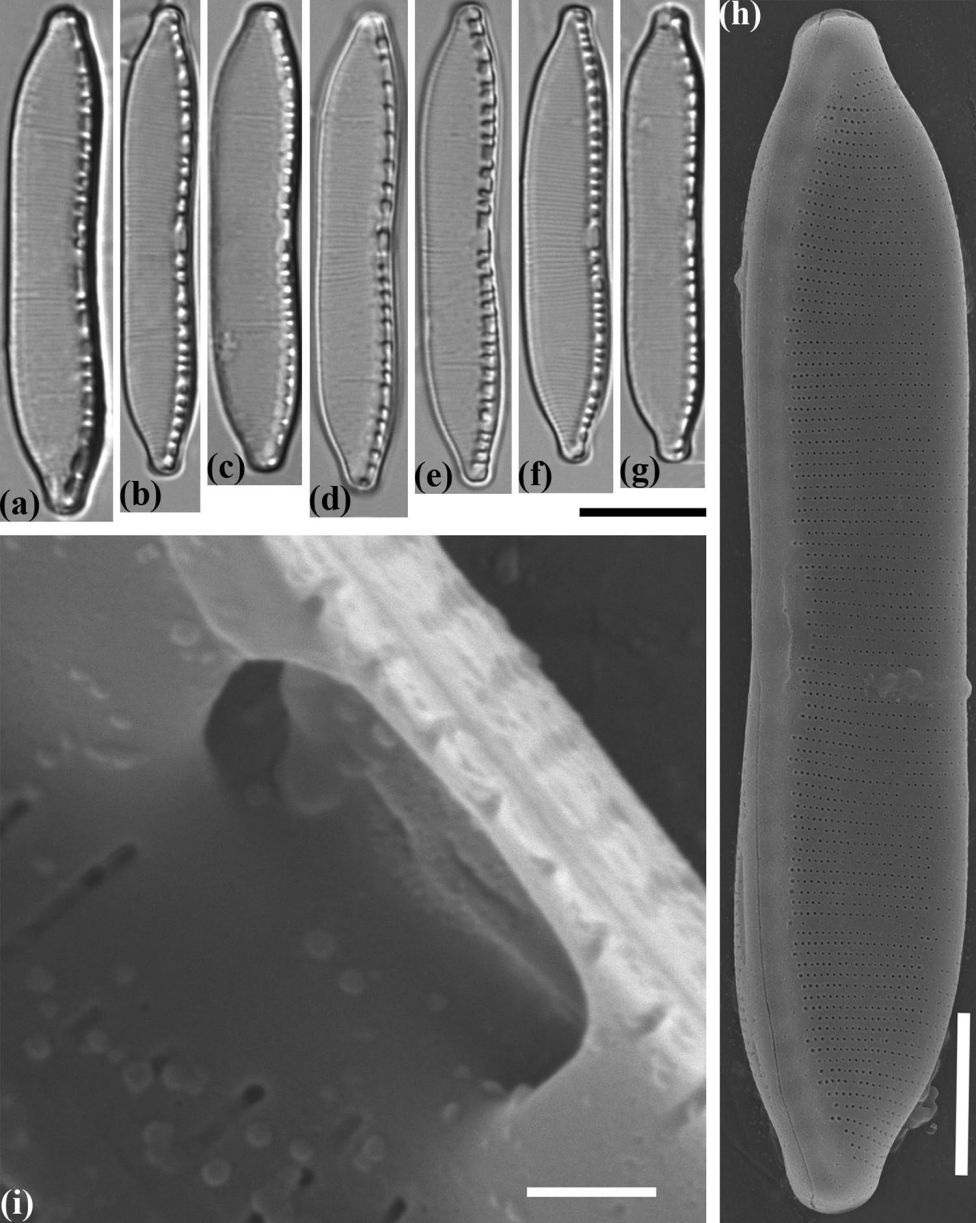
Valve morphology of the strain *Hantzschia belgica* (Sterre3)a. (**a**–**c**) Light micrographs of the natural population. (**d**–**g**) Light micrographs of monoculture. (**h**) Scanning electron micrograph, exterior of valve. (**i**) Scanning electron micrograph, interior of valve at valve center, showing bent to opposite directions of the central raphe endings. Scale bars (**a**–**g**) = 10 µm, (**h**) = 5 µm, (**i**) = 0.5 µm.

##### Holotype

Slide no. CSO86/(Sterre3)a (Holotype represented by Fig. 8) in the collection of Universiteit Gent.

##### Type locality

Soil, Gent, Oost-Vlaanderen (East Flanders), Belgium. N51°01′38.6″ E3°42′58.4″, 21.11.2007, collected by Caroline Souffreau.

##### Registration

https://phycobank.org/102520.

##### Diagnosis

Valves linear-lanceolate, weakly asymmetrical to the apical axis, with small, protracted apices. Length 36–38 µm, breadth 6–7 µm. Fibulae variable in size and spacing along the ventral margin. Central fibulae more widely spaced than the others, 8–9 in 10 µm. Striae 22–23 in 10 µm.

##### Ultrastructure

Raphe not continuous and eccentric, placed on the valve mantle for the entire length. In inside view central raphe endings curved in opposite directions. Externally, areolae are rounded; internally they are rectangular in shape. Areolae fine, 50–55 in 10 µm.

##### Etymology

The species named according to the region where it was found.

##### Molecular analysis

In the phylogenetic tree based on the 28S rDNA and *rbc*L genes, *H. belgica* (Sterre3)a occupies a separate position, sister to *H. amphioxys* plus *H. stepposa* clade (Fig. 2). These three taxa are closely related, with high statistical support (LB 100; PP 100) and represent a group of *Hantzschia* species, in which the central raphe ends on the inner side of the valve and are deflected in opposite directions.

#### Hantzschia amphioxys (Ehrenberg) Grunow emend. Maltsev et Kulikovskiy (Fig. 9)

**Figure 9:**
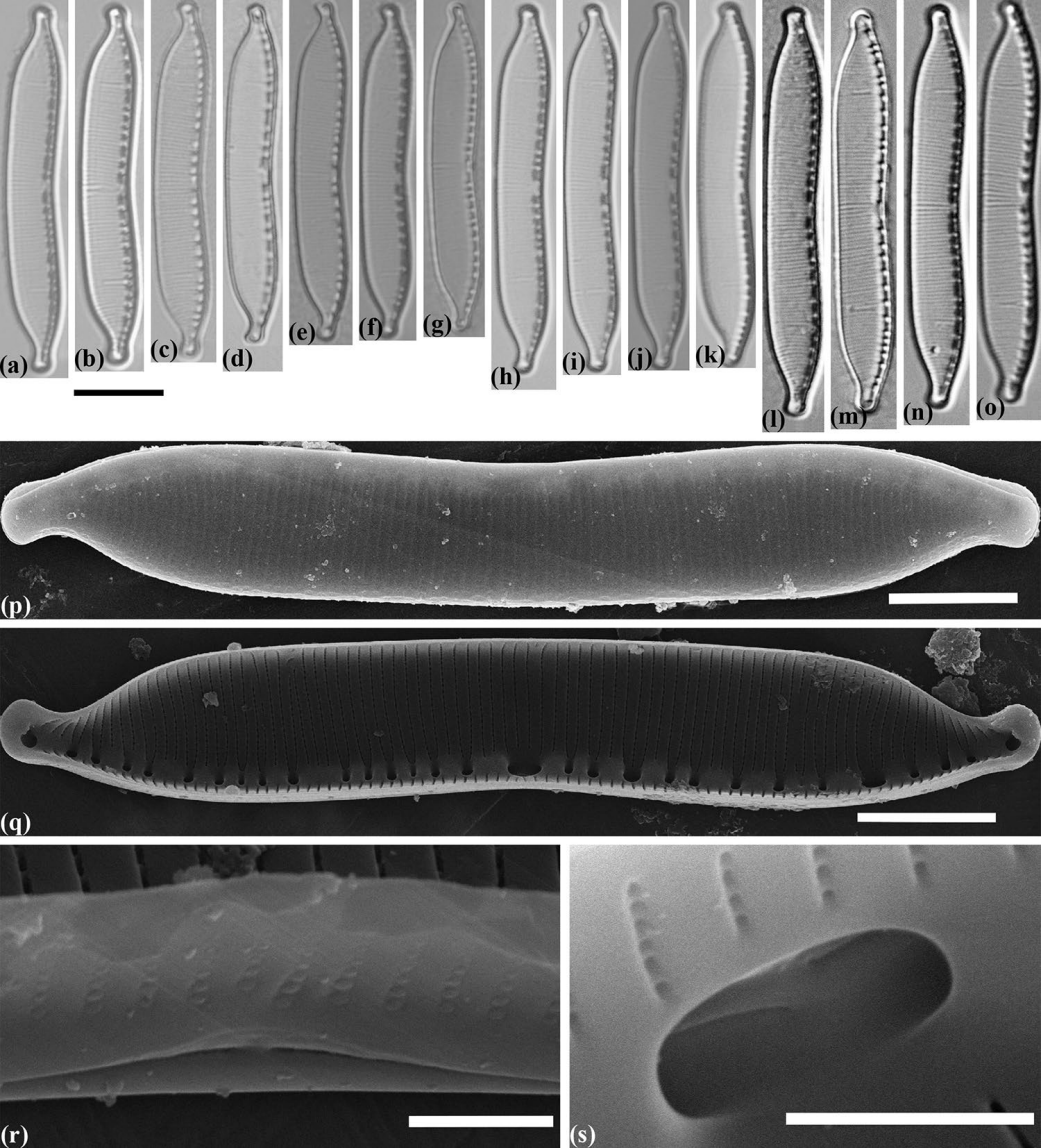
Valve morphology of representatives from the different strains *Hantzschia amphioxys*. (**a**–**o**) Light micrographs. (**a**–**c**) *H. amphioxys* MZ–BH15. (**d**–**g**) *H. amphioxys* MZ–BH8. (**h**–**k**) *H. amphioxys* MZ–BH9. (**l**–**o**) *H. amphioxys* MZ–BH14. (**p**) Scanning electron micrograph of *H. amphioxys* MZ–BH9, exterior of valve. (**q**) Scanning electron micrograph of *H. amphioxys* MZ–BH8, interior of valve. (**r**) Scanning electron micrograph of *H. amphioxys* MZ–BH8, exterior of valve at valve center, showing almost straight the central raphe endings. (**s**) Scanning electron micrograph of *H. amphioxys* MZ–BH9, interior of valve at valve center, showing bent to opposite directions of the central raphe endings. Scale bars (**a**–**o**) = 10 µm, (**p**,**q**) = 5 µm, (**r**,**s**) = 1 µm.

##### Morphology

Valves linear-lanceolate, with apices narrowly protracted, deflected dorsally. Fibular approximately of similar size and equally-spaced, with the two central fibulae more separate from one another as compared to others. Length 36–46 µm, breadth 4.8–6.5 µm. Fibulae 7–10 in 10 µm. Striae 21–24 in 10 µm.

##### Ultrastructure

Raphe not continuous. In outside view central raphe ends slightly curved to the same side (Fig. 9r). In inside view central raphe endings distinctly curved in opposite directions (Fig. 9s). Number of areolae are variable in different strains: from 40–50 in MZ–BH9 to 50–60 in MZ–BH14. Areolae range from 40 to 60 in 10 µm between the strains investigated.

##### Molecular analysis

In the phylogenetic tree, all the *H. amphioxys* strains isolated by us, as well as the sequences taken from BOLDSYSTEMS, form a separate clade (Fig. 2). This clade has several prominent subclades, formed with high statistical support. The first subclade (SCI) is represented by two strains of *H. amphioxys* (Sterre1)e and (Sterre3)c, isolated from soil in the Gent (Belgium) region^16^. The second subclade (SCII), which is sister to SCI, is formed by three strains, also isolated from Gent (Belgium), which are 2–5 µm shorter than the valves compared to the first group, one of our strains, MZ–BH8 and the epitype *H. amphioxys* D27_008. A separate monophyletic group within *H. amphioxys* has the other two subclades. The third subclade (SCIII) consists of three strains of *H. amphioxys* isolated from Schirmacher Oasis (Antarctica)^16^. The fourth subclade (SCIV) is represented by our three strains: MZ–BH9, MZ–BH14 and MZ–BH15 from widely separated habitats in Russia and Ukraine.

##### Ecology and distribution

The four *H. amphioxys* MZ–BH8, MZ–BH9, MZ–BH14 and MZ–BH15 strains we have identified belong to different, geographically distant, soil populations (Fig. 1, Table 2). Strain MZ–BH8 and MZ–BH9 were isolated from soil in mixed deciduous forests. Strain MZ–BH14 was isolated from the fermentation sub-horizon of forest litter in the *Pinus pallasiana* (D. Don) Holmboe planting. In the algae flora of this litter, *H. amphioxys* is one of the dominants^36^. Strain MZ–BH15 was isolated from the forest floor in the *Pinus sylvestris* L. plantation.

### Biogeography

The *H. abundans* clade contains strains isolated in different regions of Belgium: (Sterre6)e and (Sterre6)f from Gent, (Wes1)b and (Wes3)b from De Panne^16^, as well as our strains from Zhytomyr, Kyiv and Kherson regions of Ukraine (Fig. 2). Clade *H. attractiva* plus *H. pseudomongolica* (“*Hantzschia* sp. 2” of Souffreau et al.^16^) represents rather remote populations: *H. attractiva* MZ–BH11 was isolated in the Yaroslavl region of Russia, and strains *H. pseudomongolica* (Mo1)a, (Mo1)e, (Mo1)m—in the mountain region of Kangai-Nuruu Mongolia. However, the presence of a stable phylogenetic connection between these populations undoubtedly underlines their close relationship. Also quite interesting is the relationship between the Canadian strains of *Hantzschia* sp. 1 (Ban1)b, (Ban1)e, (Ban1)f, (Ban1)h and Mongolian *Hantzschia* sp. 2 (Mo1)h, (Mo1)l. As for the clade of *H. amphioxys*, it represents the most distant populations from the Antarctic, Belgium, Germany, Russia and Ukraine. Moreover, the selected subclades do not always correspond to the region: the SCI subclade includes two Belgian strains (Sterre1)e and (Sterre3)c; SCII subclade is formed by three Belgian strains (Sterre4)a (Sterre4)b, (Sterre6)b, one Berlin strain D27_008 and strain MZ–BH8 from the Moscow Region (Russia); SCIII subclade consists of three Antarctic strains SCHIR S11, SCHIR S15, SCHIR S16; SCIV subclade is represented by our three strains: MZ–BH9 (Kursk region, Russia), MZ–BH14 and MZ–BH15 (Zaporizhia region, Ukraine). Due to the absence of distinct differences in morphology and ultrastructure between representatives of the subclade SCI–SCIV (Fig. 2) we don’t distinguish new species which may correspond to the separate monophyletic subclades within *H. amphioxys*.

Analysis of the distribution of the studied strains showed the presence of two trends in the biogeography of *Hantzschia* species. The first one shows that within the genus there are a number of widely distributed species; the second one shows that there are also species with a probably limited distribution (see also for *Planothidium* by Jahn et al.^12^). Neither *H. abundans*, nor *H. amphioxys* had strains that are more closely related to local endemics, and given the habitats confirmed in our study, they are species with wide distribution. Also, *H. parva* can be considered as widely distributed, two strains of which were isolated from habitats distant more than 400 km apart. The second trend may indicate the presence of species with a limited distribution: *H. stepposa*, *H. abundans* (Tor3)c, and two strains of *H.* cf. *amphioxys* (Sterre1)f, (Sterre1)h. However, the question of endemism of these species has to be rethought after revising the characteristics of the morphology and ultrastructure of phylogenetically close or new strains of *Hantzschia*. Our study has shown that samples of *H. amphioxys* strains can contain both *H. amphioxys* sensu stricto itself and a number of pseudocryptic taxa. When studying the biogeography of individual taxa, it is necessary to take into account the seasonal characteristics of their vegetation. We have shown, for example, that *H. amphioxys* as a dominant of all communities in the coniferous and white acacia litter in the spring, and in the summer—only in the white acacia. In the fall, *H. amphioxys* falls out of the dominant complex and may be absent in samples^46^.

Today, there is no single and universal morphological or ultrastructural feature that would characterize the genus *Hantzschia* and the most effective way to verify membership to this genus is with a molecular phylogeny^47^. Considering this, the data obtained here for 13 new soil strains of *H. amphioxys* and 6 related species not only significantly complement the previously obtained material^16^, but also represent a significant step in understanding the diversity and biogeography of cryptic and pseudocryptic taxa in the genus *Hantzschia*.

## Materials and methods

Diatom samples of *Hantzschia* were collected in 2014–2015 from different soil and forest floor horizons in the territory of steppe, meadow and forest biogeocenoses of Eurasia (Fig. 1, Table 2). The forest floor samples were taken at a distance of 1.0–1.5 m from tree trunks in the areas without large branches and accumulated bark from the A01 (fresh debris), A02 (0–5 cm, decomposed debris with partially preserved initial structure of some components), and A03 (5–10 cm, strongly decomposed debris) sub-horizons. In total, we studied 20 soil samples, and from these 13 strains of *Hantzschia amphioxys* sensu lato were isolated. The isolated strains were deposited in the culture collection of Molecular systematics of aquatic plants laboratory at К.A. Timiryazev Institute of Plant Physiology RAS. The cultivation of the strains was done as follows: a subsample of each collection was added to a WC liquid medium^48^. Monoclonal strains were established by micropipetting single cells under the inverted microscope Zeiss Axio Vert. A1. Nonaxenic monocultures of the algae were cultivated in the liquid WC medium in Petri dishes with alternating 12-h light and dark photoperiod with 70 μmol photons m^−2^ s^−1^ light intensity. Strains were analyzed after one month of culturing.

Parts of the original strains by Souffreau et al.^16^ such as *Hantzschia* sp. 1 (Ban1)e and (Ban1)f, *Hantzschia* sp. 2 (Mo1)a, (Mo1)e and (Mo1)m, *Hantzschia* sp. 3 (Sterre6)f, *H*. *amphioxys* SCHIR_S11, SCHIR_S16, (Sterre1)e, (Sterre3)c and (Sterre4)b, *H*. cf. *amphioxys* (Sterre3)a were provided by Ghent University (Belgium) and used for morphological studies with taxonomic revisions.

Samples and strains for light microscopy and scanning electron microscopy investigations were processed by means of a standard procedure involving treatment with 10% HCl and concentrated H_2_O_2_. The material was washed with distilled water. Permanent diatom preparations were mounted in Naphrax. LM observations of the natural population and monocultures were performed using a Zeiss Axio Scope A1 microscope (Germany) equipped with oil immersion objective (× 100/n.a 1.4, DIC). The ultrastructure of the valve was examined with the JSM-6510LV (Japan) scanning electron microscope^49^. Cell measurements were based on at least 50 individuals and expressed as minimum and maximum values in the taxonomic description.

The sample selected and designated as lectotype was from the Labrador Peninsula (sample Nr. 1780) stored in the Ehrenberg collection, Museum für Naturkunde, Leibniz-Institut für Biodiversitäts- und Evolutionsforschung an der Humboldt Universität zu Berlin (BHUPM) with the mica-reference number 250502b blue. Additionally, in the Algae Collection, Botanischer Garten und Botanisches Museum Berlin-Dahlem, Freie Universität Berlin (B) an isolectotype (B 40 0040891) was selected, and the strain D27_008 was designated as epitype with its V4 domain of 18S rDNA already published^20^; *rbc*L and 28S rDNA sequences of *Hantzschia amphioxys* strain D27_008 have been submitted to NCBI in this study (for methods of extraction see Abarca et al.^50^).

Total DNA of monoclonal cultures was extracted using InstaGene Matrix according to the manufacturer’s protocol. Fragments of 18S rDNA (380 bp, including V4 domain), partial 28S rDNA gene including the D1-D3 region (753 bp) and partial *rbc*L plastid gene (1032 bp) were amplified using primers from Zimmerman et al.^51^ for 18S rDNA fragments, from Jo et al.^52^ for 28S rDNA fragments and from Ruck and Theriot^42^ for *rbc*L fragments. The *rbc*L and 28S rDNA sequences were used for phylogeny reconstruction. V4 domains were used for the step–by–step comparison of the 18S rDNA sequences of our strains and other *Hantzschia* strains.

Amplifications of the 18S rDNA fragments, partial 28S rDNA and *rbc*L genes fragments were carried out using the premade mix ScreenMix (Evrogen, Russia) for the polymerase chain reaction (PCR). The conditions of amplification for 18S rDNA fragments were: an initial denaturation of 5 min at 95 °C, followed by 35 cycles at 94 °C for denaturation (30 s), 52 °C for annealing (30 s) and 72 °C for extension (50 s), and a final extension of 10 min at 72 °C^49^. The conditions of amplification for partial 28S rDNA were: an initial denaturation of 5 min at 95 °C, followed by 45 cycles at 94 °C for denaturation (30 s), 62 °C for annealing (30 s) and 72 °C for extension (80 s), and a final extension of 10 min at 72 °C. The conditions of amplification for partial *rbc*L were: an initial denaturation of 5 min at 95 °C, followed by 45 cycles at 94 °C for denaturation (30 s), 60 °C for annealing (30 s) and 72 °C for extension (80 s), and a final extension of 10 min at 72 °C^49^.

The resulting amplicons were visualized by horizontal agarose gel electrophoresis (1.5%), colored with SYBR Safe (Life Technologies, United States). Purification of PCR products was carried out with a mixture of FastAP, 10 × FastAP Buffer, Exonuclease I (Thermo Fisher Scientific, USA) and water. 18S rDNA fragments and partial 28S rDNA and *rbc*L genes were decoded from two sides using forward and reverse PCR primers and the Big Dye system (Applied Biosystems, USA), followed by electrophoresis using a Genetic Analyzer 3500 sequencer (Applied Biosystems, USA)^49^.

Editing and assembling of the consensus sequences were carried out by comparing the direct and reverse chromatograms using the Ridom TraceEdit program (ver. 1.1.0) and Mega7^53^. Newly determined sequences and DNA fragments from 27 other *Hantzschia* species, which were downloaded from GenBank and BOLD (taxa, strain numbers and Accession Numbers are given in Supplementary Table S3), were included in the alignments.

The nucleotide sequences of the 28S rDNA and *rbc*L genes were aligned separately using the Mafft v7 software and the E-INS-i model^54^. For the protein-coding sequences of the *rbc*L gene, we checked that the beginning of the aligned matrix corresponded to the first position of the codon (triplet). The resulting alignments had lengths of 752 (28S rDNA) and 1033 (*rbc*L) characters and can be found in the Supplementary Alignment S4.

The data set was analyzed using Bayesian inference method with posterior probability implemented in Beast ver. 1.10.1.^55^ to construct phylogeny. For each of the alignment partitions, the most appropriate substitution model was estimated using the Bayesian information criterion (BIC) as implemented in jModelTest 2.1.10^56^. This BIC-based model selection procedure selected the following models, shape parameter α and a proportion of invariable sites (pinvar): TrNef + I and pinvar = 0.8330 for 28S rDNA; F81 + I and pinvar = 0.9030 for the first codon position of the *rbc*L gene; JC for the second codon position of the *rbc*L gene; TPM1uf + G and α = 0.8100 for the third codon position of the *rbc*L gene. We used the HKY model of nucleotide substitution instead of TrNef, F81, JC and TPM1uf given that they were the best matching model available for Bayesian inference method. A Yule process tree prior was used as a speciation model. The analysis ran for 10 million generations with chain sampling every 1000 generations. The parameters-estimated convergence, effective sample size (ESS) and burn-in period were checked using the software Tracer ver. 1.7.1.^55^. The initial 25% of the trees were removed, the rest retained to reconstruct a final phylogeny. The phylogenetic tree and posterior probabilities of its branching were obtained on the basis of the remaining trees, having stable estimates of the parameter models of nucleotide substitutions and likelihood. ML analysis was performed using the program RAxML^57^. The nonparametric LB analysis with 1000 replicas was used. As there are no outgroup taxa included in our analysis, we chose an unrooted tree design. The statistical support values were visualized in FigTree ver. 1.4.4 and Adobe Photoshop CC (19.0).

## Supplementary Information

Supplementary Tables.
Supplementary Table S1 Percent similarity (p-distance) matrix of 40 Hantzschia strains on the basis of the partial 28S rRNA gene (752 bp).
Supplementary Table S2 Percent similarity (p-distance) matrix of 40 Hantzschia strains on the basis of the partial ribulose-1,5-bisphosphate carboxylase, large subunit (344 amino acids).
Supplementary Table S3 Taxa and DNA sequence data used in phylogenetic analysis.

Supplementary Information.
Supplementary alignment 1 Alignment file of 40 partial rbcL and partial 18S rDNA sequences of 1785 characters, in txt format.
