# Publisher Correction: Biogeography of the cosmopolitan terrestrial diatom *Hantzschia amphioxys* sensu lato based on molecular and morphological data

**DOI:** 10.1038/s41598-021-89955-1

**Published:** 2021-05-07

**Authors:** Yevhen Maltsev, Svetlana Maltseva, John Patrick Kociolek, Regine Jahn, Maxim Kulikovskiy

**Affiliations:** 1 К.A. Timiryazev Institute of Plant Physiology RAS, IPP RAS, Moscow, Russia 127276; 2 Museum of Natural History and Department of Ecology and Evolutionary Biology, University of Colorado, Boulder, CO 80309 USA; 3 Botanischer Garten Und Botanisches Museum Berlin, Freie Universität Berlin, Königin-Luise-Str. 6-8, 14195 Berlin, Germany

Correction to: *Scientific Reports* 10.1038/s41598-021-82092-9, published online 19 February 2021

The original version of this Article contained errors. Legends for Table 1 and Table 2 were reversed.

Table 1 legend "List of all strains examined in this study with their GenBank accession numbers (BOLD ID). Geographic position and description of sample sites indicated."

now reads: “Comparison of species and strains of *Hantzschia* from studied material with similar taxa."

Table 2 legend "Comparison of species and strains of *Hantzschia* from studied material with similar taxa."

now reads: "List of all strains examined in this study with their GenBank accession numbers (BOLD ID). Geographic position and description of sample sites indicated."

These errors have now been corrected in the PDF and HTML versions of the Article.

